# Can Off-Training Physical Behaviors Influence Recovery in Athletes? A Scoping Review

**DOI:** 10.3389/fphys.2019.00448

**Published:** 2019-04-30

**Authors:** Pascal Izzicupo, Angela Di Baldassarre, Barbara Ghinassi, Felipe Fossati Reichert, Eduardo Kokubun, Fábio Yuzo Nakamura

**Affiliations:** ^1^Department of Medicine and Aging Sciences, G. d'Annunzio University of Chieti and Pescara, Chieti, Italy; ^2^SuperiorSchool of Physical Education, Federal University of Pelotas, Pelotas, Brazil; ^3^Department of Physical Education, São Paulo State University (UNESP), Rio Claro, Brazil; ^4^College of Healthcare Sciences, James Cook University, Townsville, QLD, Australia; ^5^Associate Graduate Program in Physical Education UPE/UFPB, João Pessoa, Paraíba, Brazil

**Keywords:** non-exercise activity, physical activity measurement, sitting interruptions, screen time behavior, athletes' health and life, accelerometry, physical activity questionnaires, sedentary behavior

## Abstract

Recently, the attention on recovery in sport increased enormously although there is lack of scientific evidence on the role of lifestyle in terms of movement [i.e., physical behaviors (PBs)], apart from sleep. Few studies assessed physical activity (PA) and sedentary behavior (SB) in athletes. The aims of this scoping review were to answer to the following scientific questions: (1) How active/inactive are competitive athletes out of training? (2) Do off-training PBs affect recovery, performance, and health? (3) What strategies can be implemented to improve recovery using off-training PBs, apart from sleep? From 1,116 potentially relevant articles, nine were eligible for inclusion in this review. The main issues identified were related to the heterogeneity concerning the types of sports, age category, gender, competitive level, sample size, and instruments/devices adopted, the paucity of studies investigating the effects of PBs while awake on recovery, and the lack of experimental designs manipulating PBs while awake to accelerate recovery. Furthermore, PA and SB domains were rarely investigated, while no research articles focused on the combined effect of 24-h PBs. Eight out of nine studies measured PA, seven SB, and two included sleep. Three studies included training practice into PA measurement by the means of accelerometry. Overall, almost the totality of the athletes achieved recommended PA levels although they sustained prolonged SB. In conclusion, more descriptive researches are needed in different athletic populations and settings. Furthermore, experimental designs aimed at investigating the effects of PBs manipulation on recovery and the putative mechanisms are encouraged.

The attentionon recovery in sport has increased enormously in the last years and, nowadays, it is almost as important as training for performance and sport success. A plethora of recovery strategies has been developed in the last years including active and passive recovery, cold-water immersion, compression garments, massage and many others, but some of them lack scientific evidence to support their use (McGuigan, 2017). Surprisingly, although a healthy lifestyle is considered a key factor for athletic recovery, practitioners, and sport scientists often overlook this aspect except for sleep, which has attracted a widespread attention in the last years (Costa et al., 2018; Nedelec et al., 2018; Vitale et al., 2019a). On the other hand, the amount of evidence on the role of an active lifestyle for health is enormous and various global and national guidelines and updates have been developed during years on the type and amount of physical activity (PA) recommended to improve and maintain health and to prevent and manage chronic diseases, in different populations (Ministry of the Education and Culture Finland, 2016; Graf et al., 2017; Pfeifer and Geidl, 2017; Mottola et al., 2018; Piercy et al., 2018). Physical activity positively affects cardiorespiratory fitness, muscular strength and endurance, blood lipids and glucose levels, body composition, balance and coordination, and mental health and well-being. On the other hand, sedentary behavior (SB) and the lack of PA affect health and well-being, negatively acting at all the above-mentioned levels. According to the recent and more comprehensive definition of PA proposed by the DEDIPAC-KH research team, it consists of any bodily movement produced by skeletal muscles that results in energy expenditure and this may be unstructured and everyday life activity, exercise that includes prearranged, deliberate, and repetitive activity and grassroots sports and competitive sports (Condello et al., 2016). Unstructured and everyday life activity is also defined spontaneous physical activity (SPA), namely every daily PA excluding any volitional exercise (Garland et al., 2011). SB is defined as any waking behavior characterized by an energy expenditure ≤1.5 metabolic equivalents (METs), while in a sitting, reclining, or lying posture. Consequently, physical inactivity is considered the lack of meeting the recommended levels of PA and not a synonymous of SB (Tremblay et al., 2017a). Collectively these terms, together with sleep, are named 24-h movement behaviors (McGregor et al., 2018) or physical behaviors (PBs) (Freedson, 2018). The World Health Organization (WHO) recommends adults to perform at least 150 min/week of moderate-intensity physical activity (MPA) or 75 min/week of vigorous physical activity (VPA), or any combination of both intensities that meets the recommended amount. Notably, it recognizes the importance of sitting less, though no quantitative key guideline for sitting time or how to break up sitting duration are proposed (Piercy et al., 2018). Furthermore, 24-h movement guidelines have been recently published for children in different countries (New Zealand Ministry of Health, 0000; Tremblay et al., 2016, 2017b; Okely et al., 2017) and it is very probable that the same format will be adopted for future recommendations (Chaput et al., 2014). The aforementioned recommendations are considered the minimum amount of PA to obtain noticeable benefits for health. Additional health benefits are gained by doing physical activity beyond the equivalent of 300 min/week of MPA (Piercy et al., 2018). On the other hand, athletes are active “*de facto*” because they exercise regularly and athletes from most sports disciplines exceed those recommendations. Indeed, the mortality risk has been reported to be lower in elite athletes compared to non-athletes, particularly in endurance athletes (Garatachea et al., 2014; Kettunen et al., 2015; Lemez and Baker, 2015). However, the amount of time spent in training represents a reduced part of the full day and at present the majority of studies focus their attention only on the effects of sleep on recovery and performance, while PA and SB, during almost two-thirds of the day, have been investigated in a restricted number of studies. It is well-known that PBs while awake affect both bodily systems (e.g., vascular system, endocrine system, and immune system) and metabolic pathways (i.e., glucose and lipids), as well as systemic and local inflammation, mood, fatigue, and cognition (Pedersen and Saltin, 2015). However, there is a lack of evidence in scientific literature about the repercussions of off-training PBs in competitive athletes on recovery, training adaptations and performance, as well as on short and long-term career. Given the above considerations and the recent progresses in accelerometry micro-technology (Freedson, 2018), an overview of the studies describing off-training PBs in competitive athletes throughout the days, not only during sleep is warranted. For this purpose, considering the heterogeneity and the complex nature of the topic, and considering that it has not yet been comprehensive revised, a scoping review is appropriate. The aim of a scoping review is to identify knowledge gaps, scope a body of literature, clarify concepts or to investigate research conduct in emerging body of evidence and make recommendations for future researches. It answers to broader questions beyond those related to the effectiveness of an intervention or treatment (Peters et al., 2015). Then, the aim of this scoping review article is to provide an overview of the body of literature describing PBs while awake in competitive athletes and their effects on recovery, training adaptation, and performance, as well as on related factors (e.g., lactate clearance). This article aims to answer to the following scientific questions: (1) How active/inactive are competitive athletes out of training and do they differ on the basis of competitive level, age categories, and particular settings (e.g., home-based training, training camp, traveling, and tournament participation)? (2) Do off-training PBs affect recovery, performance, health, physical fitness, and career in competitive athletes and by means of which mechanisms? (3) What strategies can be implemented in competitive athletes to improve recovery using off-training PBs, apart from sleep? For the purpose of clarification, a competitive athlete is considered in this scoping review article a highly trained individual who engages in regular organized physical training within a particular sporting discipline and competes at county or national or international level (Sharma, 2003). This scoping review introduce the 3ST (Sleep, Sedentary behavior, Spontaneous physical activity, and Training) project which proposes to (1) provide a complete and detailed description of PBs of competitive athletes using an observational design; (2) explore associations between competitive athletes' PBs and physiological/psychological markers of health, performance, and recovery, as well as describe the mechanisms that regulate such associations and; (3) test the effectiveness of interventions (e.g., education and therapies) to improve competitive athletes' lifestyles and habits, aiming at optimizing training adaptation, recovery, and performance. Information gathered for this scoping review and the potential future results of the 3ST project could be useful for sports scientists, practitioners, and, ultimately, athletes, as it may help in the understanding of the conceptual and methodological gaps in the current health-enhancing and training recovery literature. Additionally, this review and the associated 3ST project may inspire the design of high-quality studies in the field across different athletic populations and the various forms of training-related demands.

## Methods

### Search Strategy

A three-step searchstrategy was utilized in this review up to February 8, 2019. An initial limited search of MEDLINE was undertaken using the terms: physical activity, sedentary behavior, athletes, and recovery, followed by an analysis of the text word contained in title and abstract and of the index terms used to describe the articles. A second search of MEDLINE, ISI Web of Science, and Scopus was undertaken using all the identified keywords and index terms in the following four categories: population, assessment, comparison/subgroup, outcome/phenomenon of interest (Table 1). Search syntax is available in Supplementary Material. Thirdly, the reference list of all the identified manuscripts were searched for additional studies. Considering the broad international scope of the current review, no restrictions about language, type of study, and journal categories were applied. Furthermore, no age and gender criteria were imposed according to the objective of investigating the role of different group and settings on PBs. Based on the relevant recent definition of SB (Sedentary Behaviour Research Network., 2012) date criteria was limited to the articles published during and after 2012.

**Table 1:** Identified terms for the search strategy according to the categories of interest.

**Population**	**Assessment**	**Comparison/subgroup**	**Outcome/phenomenon of interest**
Player^*^ Athlete^*^ Amateur^*^ Professional^*^ Elite	Self-reported time Questionnaire^*^ Acceleromet^*^	Highly trained Injur^*^ Recreational Young adult^*^ Master^*^ Young athlete^*^ Adolescent^*^ Youth Non-athlet^*^	Sitting Physical activit^*^ Sedentar^*^ Recovery Performance

*The identified keywords were searched according to the four categories in the table to provide a better structure to the search strategy*.

* *indicates end-truncation in search strategy*.

### Inclusion Criteria

Included studies were those that incorporated competitive athletes without restriction concerning age, gender, and competitive level. Since the aim of this scoping review is to map the knowledge concerning PBs in athletes, studies including those behavior as primary or secondary outcomes were included. Studies concerning sleep were considered only when sleep measurement and assessment were accompanied by some other PBs while awake.

### Exclusion Criteria

Studies including only recreational sport participants were not included. Furthermore, no studies were considered in which it was not possible to infer if participants were competitive or not.

### Data Extraction

From potentially relevant articles, generic information (e.g., author name, journal name, and year) and abstract were saved for the analysis. Two independent researchers independently processed all data, with one extracting information and the other verifying. Quantitative data (e.g., sample size, period of assessment) and qualitative data (e.g., phenomena of interest, setting, authors conclusions) were extracted.

### Eligible Articles

**Figure 1:**
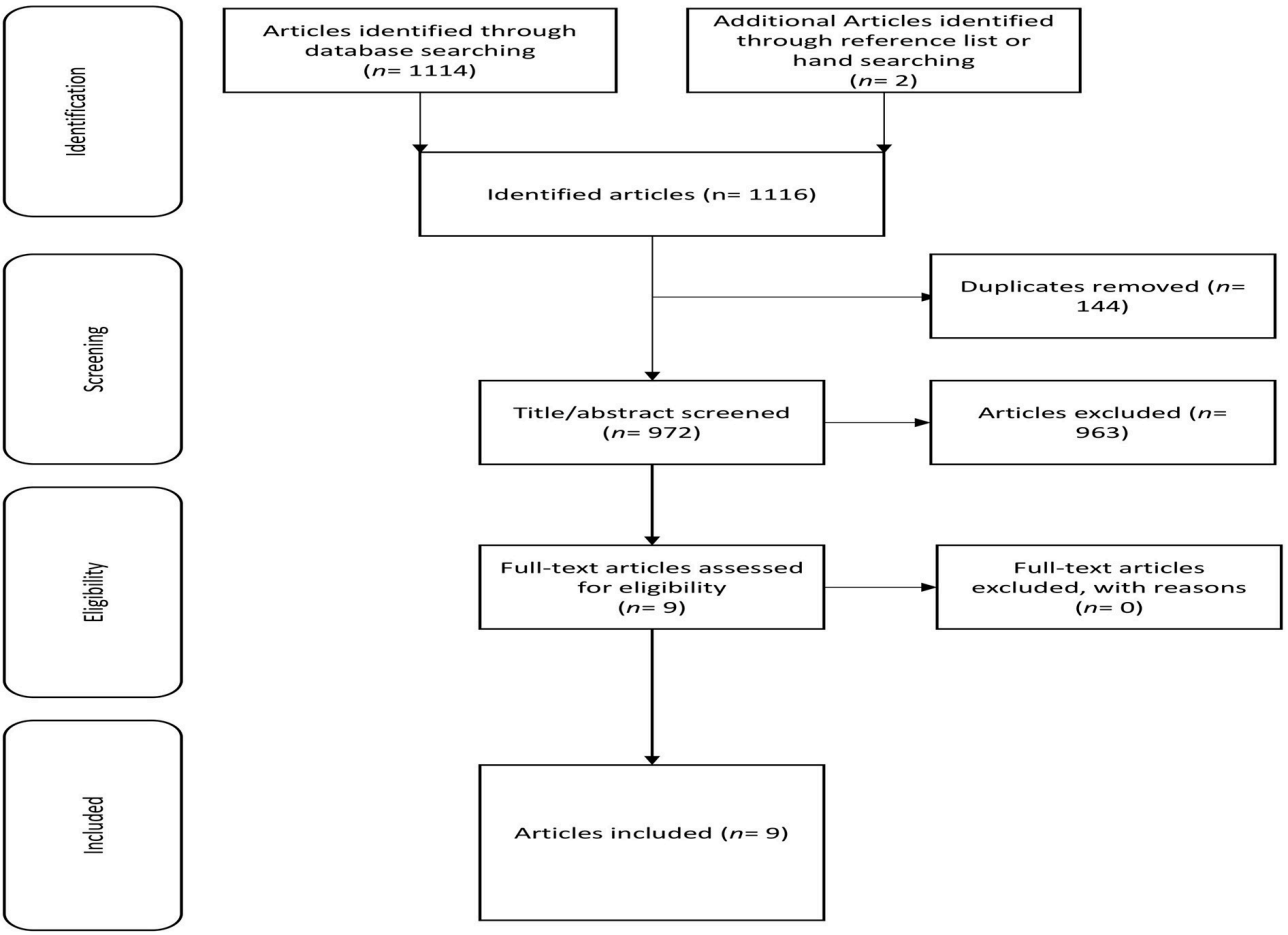
Flow chart illustrating the different phases of the search and study selection.

From 1,116 articles, 1107 were excluded (Figure 1), leaving 9 eligible for data extraction.

## Results

### General Information

**Figure 2:**
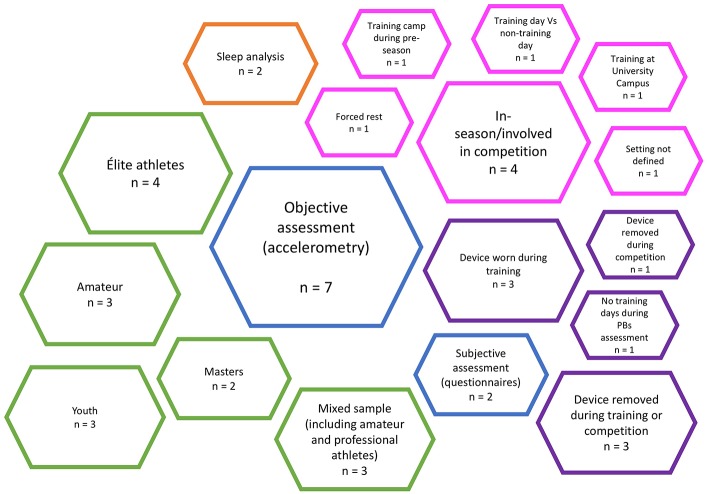
Map of the identified qualitative items by number of studies. Blue, method of assessment; purple, inclusion/exclusion of training/competition during PBs assessment; orange, sleep analysis; magenta, setting, phase of the season; green, sample.

**Figure 3:**
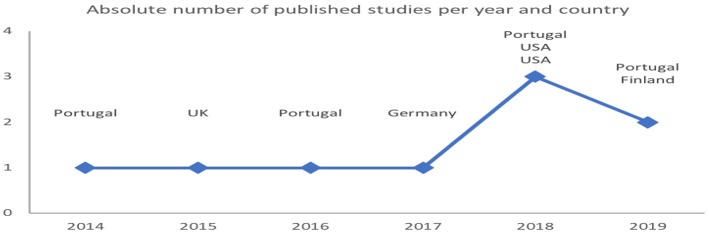
Eligible articles published per year and country.

Items considered for data extraction and the identified categories are summarized in Figure 2. All the nine eligible articles were written in English and published in 9 different journals, from 5 countries, between 2014 and 2019 (Figure 3). One study compared PA and SB between athletes and non-athletes (Clemente et al., 2016), and two studies correlated PBs with some physiological/psychological markers, in competitive athletes (Júdice et al., 2014; Sufrinko et al., 2018). Overall, there is substantial heterogeneity in the selected studies concerning the types of sports, age category, gender, competitive level, sample size, and instruments/devices adopted to assess PBs (Table 2). Júdice et al. (2014) performed the study in a crucial time of the competitive season (e.g., the last days before engaging in an international competition), Weiler et al. (2015) during a week of the competitive season, Clemente et al. (2016) assessed university athletes the majority of which lived in rented flats or in the campus hostels with a small home-to-university distance, but they did not give information about the competitive period, as well as McCracken and Dogra (2018); Sperlich et al. (2017) performed the study during a preparation training camp in the pre-season, while Exel et al. (2018, 2019) during a typical week of the competitive season. Finally, Sufrinko et al. (2018) performed the study during recovery after concussion. Not all the studies reported the monitoring device sampling frequency, as well as the epoch length at which data were collected and analyzed/collapsed (Table 3).

**Table 2:** General characteristics and main results of the selected articles.

**Authors**	**Disciplines**	**Gender**	**Competitive level**	**Sample size (num)**	**Age (years, Mean ± sd)**	**Assessed behaviors**	**Main results**
Júdice et al., 2014	Various disciplines	Males	Elite	82	21.8 ± 4.8	Sitting time	Sitting time predicts total fat and trunk fat mass independent of age, weekly training time, and residual mass but not abdominal fat. Weight-class sports is the category most responsible of this association, compared to non-weight sensitive sports and gravitational sports.
Weiler et al., 2015	Soccer	Males	Elite	25	26.8 ± 4.4	SB, LPA, MPA, VPA	The majority (79%) of post-training time of elite soccer players from an English Premier League football club is spent in sedentary activities.
Clemente et al., 2016	Various disciplines	Males and females	Amateurs and professionals	33	NA	SB, LPA, MPA, VPA, steps	Despite some statistical differences with minimal effect size (LPA for men and SB, LPA, and VPA for women), the results of this study suggested proximity between PA levels of athletes and non-athletes, mainly in the case of SB.
Sperlich et al., 2017	Rowing	Males	Elite (U23 men's national team)	11	20.0 ± 2.0	SB, LPA, MPA, VPA, sleep	Rowers display a considerable amount of time spent in sedentary pursuits (about 11.5 h/day).
McCracken and Dogra, 2018	Various disciplines	Males and females	Local to international masters	79	63.6 ± 7.2	Sitting time, MPA, VPA, PA and SB domains	Male recreational athletes spend more time in SB and less time in VPA compared to master athletes, while female recreational athlete spend less time in SB in comparison to master athletes. Although older athletes accumulate high volumes of SB, they also accumulate the suggested 60–75 min of moderate-vigorous intensity PA per day to negate the detrimental effects of sitting.
Exel et al., 2018	Various disciplines	NA	Elite (youth)	8	15.7 ± 2.0	SB, LPA, MPA, VPA, MVPA, standing, sitting, lying, sedentary breaks over 30 min	Young athletes showed different patterns of PA and SB. Most weekdays waking hours are spent in places that promote sedentarism (school and home). Some athletes still manage to balance healthy PA and SB levels and may serve as a reference.
Sufrinko et al., 2018	Various disciplines	Males and females	NA	19	15.5 ± 1.9	Bed time, sleep time, sleep efficiency, variation in total sleep time, total PA, mean PA, PA intensity	PA increases during recovery from concussion while total time in bed decreases, although total amount of sleep and sleep efficiency did not change. Both PA and sleep are associated with neurocognitive and vestibular/oculomotor outcomes.
Exel et al., 2019	Footballers and runners	Males	Footballers: local masters; Runners: recreational masters	29	43.9 ± 3.9	SB, LPA, MPA, VPA, MVPA in 10 min bouts,	Different sports determine different distributions of PA levels in adults. Amateur runners tend to higher amounts of VPA, while footballers perform higher amounts of LPA and MPA. There are no differences in terms of SB.
Ala-Kitula et al., 2019	Soccer players	Males	National	18	12.6 ± 0.3	LPA, MPA, VPA, MVPA, SB lasting at least 30 min	The amount of MVPA attained on practice days is not achieved on days without practice. On weekdays without practice the MVPA recommendations are not met. Previous PA of the same day before soccer practice has positive correlation with PA during soccer practice at several different activity levels.

*SB, sedentary behavior; PA, physical activity; LPA, light-intensity physical activity; MPA, moderate-intensity physical activity; VPA, vigorous-intensity physical activity; MVPA moderate to vigorous-intensity physical activity*.

**Table 3:** Technical specifications of the accelerometer settings of the selected articles.

**Author**	**Instrument/device**	**Days of assessment**	**Wear time**	**Body site**	**Sampling frequency**	**Epoch length**	**Metrics**	**Cut-points**
Weiler et al., 2015	GENEActiv triaxial wrist accelerometer (Activinsights Limited, Cambridge, UK)	7 consecutive days	≥500 min/day of continuous wear time during waking hours of the off-training period	Wrist (dominance not specified)	50 Hz	Not specified	Activity counts	Not specified
Clemente et al., 2016	ActiGraph accelerometer wGT3X-BT (Actigraph Corp, Shalimar, FL, USA)	Seven consecutive days	24 h/day, apart from water-based activities. Sixty minutes without activity (zero counts) was considered non-wear time and not included in the data treatment.	Wrist (dominance not specified)	Not specified	Collected at 10-s epochs, subsequently collapsed into 60-s epochs	Activity counts	SB ≤ 100 cpm; LPA = 100–1,951 cpm; MPA = 1,952–5,724 cpm; VPA ≥ 5,725.
Sperlich et al., 2017	Wrist-worn multisensory device Microsoft Band II (Microsoft Corporation, Redmond, Washington, USA)	31 consecutive days, with 21 weekdays, and 10 weekend days	≥480 min/day of continuous wear time during waking hours of the off-training period	Wrist (dominance not specified)	Not specified	Microsoft Band II stores the data of mean hourly energy expenditure online	Proprietary algorithm	Not specified
Exel et al., 2018	ActiGraph GT9X Link (Actigraph Corp, Pensacola, FL, USA)	15 consecutive days	≥600 min/day of continuous wear time during waking hours of the off-training period	Hip, on the dominant side	30 Hz	Collapsed into 60-s epochs	Activity counts	SB = 0–180 counts·15s^−1^, LPA = 181–756 counts·15s^−1^, MPA = 757–1,111 counts·15s^−1^, VPA = ≥1,112 counts·15s^−1^
Sufrinko et al., 2018	ActiGraph GT3X+, (Actigraph Corp, Pensacola, FL, USA)	20.2 ± 9.7 days	24 h/day, apart from water-based activities. Non-wear time was identified and re- moved using the Troiano algorithm in Actilife Soft- ware v6.13.3 (Actigraph Corp, Pensacola, Florida)	Non-dominant wrist	Not specified	Collapsed into 60-s epochs	Activity counts	Not specified
Exel et al., 2019	ActiGraph GT9X Link (Actigraph Corp, Pensacola, FL, USA)	7 consecutive days	9–12 h/day of waking hours; removed during water-based activities and competitions.	Dominant wrist	30 Hz	Raw acceleration data	Euclidean Norm Minus One (ENMO)	intensity-specific cut-points calculated according to Hildebrand et al., 2014
Ala-Kitula et al., 2019	Polar A300 -activity monitor (Polar Electro Oy, Finland)	9 consecutive days, including 7-week days and 2 weekend days	Minimum wear time for valid data not specified.	Non-dominant wrist	Not specified	Not specified	Proprietary algorithm	Not specified

*SB, sedentary behavior; PA, physical activity; LPA, light-intensity physical activity; MPA, moderate-intensity physical activity; VPA, vigorous-intensity physical activity*.

### Off-Training Physical Behaviors While Awake in Competitive Athletes

All the included studies, except for the one from Júdice et al. (2014) assessed PA. One study (McCracken and Dogra, 2018) assessed PA in master athletes using the short form of the International Physical Activity Questionnaire (short-IPAQ), which assesses MPA, VPA, walking, and sitting time. Six out of seven accelerometry studies categorized PA according to its intensity in LPA (1.5–3 METs), MPA (3–6 METs), and VPA (>6 METs), although not all the studies reported the cut-points used to calculate the intensity (Table 3). On the other hand, Sufrinko et al. (2018) only reported total daily vector magnitude (VM) counts, average VM counts per minute, and maximum VM counts per minute, making it difficult to categorize PA levels and to compare with other studies. Nevertheless, even among studies using the same threshold-based analysis for classifying PA by its intensity, the comparison remains difficult, due to the lack of some information on the technical specifications and the differences in instrumentation, setting, and data processing (Table 3). Overall, the recommendation on PA levels for health was met on average by all the samples of the considered studies that reported PA levels, apart from children in days without sport practice (Ala-Kitula et al., 2019). Clemente et al. (2016) and McCracken and Dogra (2018) compared university and master competitive athletes with non-athletes and recreational athletes, respectively. Despite some statistical differences with small effect size (LPA for men and SB, LPA, and VPA for women), the results of Clemente et al. (2016) suggest proximity between PA levels of athletes and non-athletes. On the other hand, male master athletes in the study from McCracken and Dogra (2018) showed significantly higher levels of VPA compared to male (age-matched) recreational athletes. Another study on male master runners and footballers (runners were recreational participants) also indicated that master athletes meet global recommendations on PA (including training time) (Exel et al., 2019). A study on young footballers compared days with soccer practice with days without soccer practice showing that in the days without training children fail to reach the national recommendations on MVPA (Ala-Kitula et al., 2019).

All the studies that assessed SB reported worrisome levels of sedentariness, regardless of using subjective or objective methods. No difference emerged when competitive athletes were compared to non-athletes (Clemente et al., 2016) or recreational masters (McCracken and Dogra, 2018). However, when considering the domains of SB, some differences were found between master and recreational athletes: male master athletes spent less time watching TV than the recreational ones and less time in hobbies that imply sitting, as well as at work and during leisure time, compared to both male recreational athletes and female master athletes. On the other hand, female master athletes spent more time sitting at work and during leisure time than female recreational athletes, although this result may be due to the higher rate of workers among master than recreational athletes. Interestingly, Exel et al. (2018) found that young athletes showed different patterns of activity (i.e., more or less active), in spite of the time spent in environments which can favor SB, such as school and home. Adult recreational runners and master footballers competing at local level showed no difference in terms of SB, although they differed in LPA, MPA, and VPA but not in MVPA cumulated in bouts of at least 10 min (Exel et al., 2019). Overall, these studies suggest that athletes have a similar SB to the general population but the majority meet PA recommendations, although results about children are matter of concern.

### Association Between PBs While Awake and Markers of Recovery, Performance, Health, and Physical Fitness, in Competitive Athletes

Júdice et al. (2014) reported that sitting time was positively associated with total and trunk adiposity while weight class sports showed higher values of sitting time, in spite of a lower amount of training, and a stronger association between sitting time and adiposity. Sufrinko et al. (2018) showed that both PA and sleep changed throughout the course of the recovery from sport-related concussion. In particular, PA intensity (i.e., maximum VM counts per minute) increased and total time in bed decreased throughout concussion recovery, without any difference in total sleep time and sleep efficiency. Furthermore, both PA and sleep were associated with clinical outcomes of recovery. Both PA and sleep were associated with clinical outcomes of recovery: visual memory scores at follow up (sub-acute phase) were linearly and positively associated with PA intensity during the first week after recovery (acute phase), while very high and very low average PA counts during the acute phase were associated with worse motor speed scores at follow up (inverted “U” relationship). In addition, PA during the first week postinjury was also associated with worse vestibular/oculomotor scores at follow up. Finally, Ala-Kitula et al. (2019) investigated the relationship between SPA preceding training and PA during training: SPA before the practices had significant positive correlations with different activity levels during practices on weekdays and on weekend days. The highest correlation was found between preceding LPA and MPA during training on the same days, on weekdays and on weekend days.

### Intervention Studies on Physical Behaviors While Awake in Competitive Athletes

No studies were found that manipulated PBs while awake with the aim of improving health, recovery, performance, training adaptations, or any other related outcomes in competitive athletes.

## Discussion

The aim of this scoping review was to answer to the following scientific questions: (1) how active/inactive are competitive athletes out of training and are there differences in their PBs on the basis of competitive level, age categories, and settings? (2) Do off-training PBs affect recovery, performance, health, physical fitness, and career in competitive athletes and by means of which mechanisms? (3) What strategies can be implemented in competitive athletes to improve recovery by using off-training PBs, apart from sleep? The main results of the present article reveal the paucity of information about off-training PBs while awake in competitive athletes, although the number of articles increased in the last years (Figure 3), as well as about the association of the PBs with physiological/psychological markers of health, performance, and recovery. Furthermore, the results of this scoping review indicate that there are no studies with an experimental design that might link off-training PBs while awake with recovery, performance, health, and physical fitness. Moreover, there are no studies that attempted to improve these aspects by means of manipulation of the off-training PBs while awake. Finally, the large variability in the eligible studies concerning the populations and the contexts, the instruments and their settings, as well as data processing procedures, make comparison and generalization of the results difficult. Notwithstanding, in the following paragraphs, a discussion is provided on the few emerging evidence and their implications regarding PBs in athletes and future directions for off-training lifestyle research in competitive athletes are indicated.

### How Active/Inactive Are Competitive Athletes out of Training?

Overall, the included studies agree on the following points: first, athletes spend too much time in sedentary pursuits, which make them comparable to the general population (Clemente et al., 2016); secondly, most of them meet PA recommendations, although the younger athletes seems to face more difficulties in doing so (Exel et al., 2018), especially in the days when they do not train (Ala-Kitula et al., 2019). In recent years, scientific studies have indicated that too much sitting is harmful for health, independent of PA. However, Ekelund et al. (2016) provided evidence suggesting that high PA levels (60-75 min of MVPA/d) are protective against the risk of death associated with prolonged sitting and TV-viewing. As athletes can easily exceed thses levels of PA, health issues should not be a matter of concern for them. However, while the results of this scoping review indicate that most of the athletes meet the international recommendations on PA, some of them such as university athletes and young soccer players (Table 4) may not reach the levels suggested by Ekelund et al. (2016) to counteract the harmful effects of SB. Notably, SB are excessive for all the populations investigated in the eligible studies of this scoping review. The only exception is represented by a subgroup of young athletes in the study of Exel et al. (2018) that showed a pattern of activity that was less sedentary and more active compared to the others, despite having been in the same environments (home and school). As suggested by the authors, this subgroup seems to show a resilience to the “sedentarigenic environments” (home and school) and researchers should investigate the reasons of such behavior because they can represent a healthy reference to the others. This is particularly important considering another result from the study of Ekelund et al. (2016): while high levels of PA seem to eliminate the increased risk of mortality associated with prolonged sitting (more than 8 h/d) they only mitigate, not eliminate the risk associated with protracted TV-viewing (more than 5 h/d), a behavior generally performed at home, suggesting that the domains of activities while sitting are as relevant as the amount of exposure to SB. In a recent study, Jones et al. (2019) showed in well-trained athletes that the use of multiple devices in the evening was associated with more perceived difficulty in falling asleep. Considering the already-known importance of good sleep in athletes and that the use of electronic devices can disturb attention and mood, which are also fundamental in sports training and competitive performance (Green et al., 2017), the investigation of the inherent relationships between training, SPA, SB, and sleep during 24 h appears of crucial importance, together with the need to study the domains of PA and SB. These behaviors take place in a *continuum* from sleep to very vigorous PA and can occur in different parts of the day. Recent studies showed that the intervention on one of those behaviors (e.g., introducing moderate-intensity physical exercise) would necessarily have consequences on the others (Izzicupo et al., 2012; Blasio et al., 2018), a phenomenon that can make efforts for improving health vain (Di Blasio et al., 2012). For this reason, several studies have investigated the combined effect of 24-h PBs on health using isotemporal and compositional data analysis techniques (Biswas et al., 2015; Colley et al., 2018; McGregor et al., 2018). This can be even more important for athletes due to the fact that they are involved, generally, in very vigorous training sessions or in mentally-demanding activity (e.g., archery), which can impact on other behaviors during the remaining time of the day. For instance, training sessions and competitions can take place during different periods of the day (e.g., morning session vs. evening session, multiple training sessions during the day, evening competition), and professional athletes can have a large amount of leisure time, while other competitive athletes are employed, possibly having different repercussions on athletes' training recovery and performance. Indeed, it has been recently shown that athletes' rest-activity circadian rhythm differs in accordance to the sport discipline (Vitale et al., 2019b). However, none of the eligible studies in this scoping review investigated the interaction effects of differently combining PBs over 24-h, but rather analyze behaviors in isolation and descriptively. It is plausible, that training load affects PBs during the remainder of the day and vice versa, as well as it is possible that sleep can be affected by training practice, daily PA and SB and their combination. However, such eventuality has not yet been studied. The only study that considered the relationship between different behaviors is the one from Ala-Kitula et al. (2019) where PA preceding soccer practice had a positive correlation with all PA intensity levels during practice. It is known that children with high PA level are active throughout the day compared to less active children (Fairclough et al., 2012). This result suggests that there might be interdependence between off-training practices and quantity and quality of movements during training. This hypothesis needs to be further addressed.

**Table 4:** Daily physical behaviors time.

**Authors**	**Sleep**	**Sedentary behavior**	**Light-intensity physical activity**	**Moderate intensity physical activity**	**Vigorous-intensity physical activity**
	**(h/day)**				
Júdice et al., 2014	NA	7.70 ± 2.70	NA	NA	NA
Weiler et al., 2015		8.34 ± 0.98	0.93 ± 0.48	1.24 ± 0.47	0.03 ± 0.06
Clemente et al., 2016					
Males	NA	12.29 ± 3.38	5.18 ± 1.91	0.88 ± 0.71	0.09 ± 0.14
Females	NA	12.17 ± 2.30	5.23 ± 1.26	0.79 ± 0.69	0.07 ± 0.11
Sperlich et al., 2017					
Week days	8.18 ± 1.24	11.63 ± 1.25	1.27 ± 1.15	0.76 ± 0.37	0.51 ± 0.44
Weekend days	8.07 ± 1.34	12.49 ± 1.10	0.67 ± 0.43	0.59 ± 0.37	0.53 ± 0.32
McCracken and Dogra, 2018					
Males	NA	4.72 ± 0.41	NA	0.64 ± 0.15	0.80 ± 0.08
Females	NA	5.63 ± 0.51	NA	0.44 ± 0.07	0.87 ± 0.12
Sufrinko et al., 2018	7.06 ± 0.0.69	NA	NA	NA	NA
Exel et al., 2019	NA	9.01 (3.25)	4.0 (2.23)	1.49 (1.4)	0.03 (0.06)
Ala-Kitula et al., 2019					
Training days	NA	NA	2.83 ± 0.85	1.3 ± 0.45	1.08 ± 0.32
Non-training days	NA	NA	3.3 ± 1.0	0.85 ± 0.43	0.42 ± 0.32

*NA, not assessed or not available. In the study of Exel et al. (2018) physical behaviors (PBs) were reported as min/h and the devices were worn only during awake time. Since it was not possible to transform accurately PBs time in h/day, the study was not included in the table. Exel et al. (2019) data are presented as median (interquantile range)*.

Screen time exposure and the domains of SB were investigated by McCracken and Dogra (2018): female master athletes exceeded the amount of screen time (more than 4 h/d) determining harmful effects for health and male master athletes were very close to this value (3.42 h/d). Then, hypothetically they can counteract the effects of too much sitting but not the effects of too much screen time. Furthermore, it must be considered that PA and SB were assessed through questionnaires in this study, which underestimate sitting time while overestimating PA, especially in older adults (Dyrstad et al., 2014). Exel et al. (2019) also indicated that master athletes meet global recommendations on PA using accelerometers. However, participants in the first study were about 20 years older than participants in the latter. In the study of Exel et al. (2019), MVPA cumulated in bouts of at least 10 min were similar between groups but runners spent more time in VPA than footballers while the latter shows higher levels of LPA and MPA in comparison to runners. However, such a difference may be attributed to differences in training routines between running and soccer, rather than to off-training PBs. Authors indeed aimed to investigate if both running and soccer are sport activities suitable for master athletes to meet PA recommendations and, for this reason, they did not separate training from non-training PA. Notably, runners were recreational participants while footballers were considered athletes although competing at local level. Nonetheless, runners showed higher VPA levels due to training routine. McCracken and Dogra (2018) also showed that master athletes spent a considering amount of time in other sitting activities at work, during leisure time or driving. This aspect may significantly differ when age category and level are considered. Scholar age athletes spent a considerable amount of time in sedentary activities at school and home, as showed by Exel et al. (2018) and this can represent a problem during the day without training practice because they can fail to reach the recommended level of PA (Ala-Kitula et al., 2019). Finally, athletes can spend a lot of time traveling due to competition schedules (Fowler et al., 2017), with few opportunities to interrupt sitting for long periods. Padilla and Fadel (2017) showed in a series of experiments that prolonged and uninterrupted sitting, a behavior common during long-haul travels or after suffering an injury, is associated with acute lower limbs dysfunction in healthy young subjects. Furthermore, prolonged and uninterrupted sitting can increase sympathetic and renin-angiotensin system activity (Young and Leicht, 2011), as well as plasma fibrinogen, hematocrit, hemoglobin, and red blood cell count, aside from a reduction in plasma volume (Howard et al., 2013). While it remains to be elucidated if an increase in blood viscosity may hinder skeletal muscle blood supply and then recovery, hypercoagulability in athletes and flight travel-associated thrombotic events (not so rare among athletes) (Bishop et al., 2017) are a matter of concern (Meyering and Howard, 2004). Although there are no previous studies that associated vascular alteration due to prolonged sitting with recovery, it is plausible that both energetic restoration and tissue repair are affected by prolonged sitting, due to the importance of muscle blood flow in these processes. Nevertheless, a comprehensive description of PBs of competitive athletes using an observational design, possibly investigating the combined effect of 24-h PBs considering their domains, is first needed to identify specific patterns of activity. Such a knowledge will be helpful in focusing research aimed to understand if and how off-training PBs can affect recovery, performance, health, physical fitness, and career in competitive athletes and through which mechanisms.

### Do Off-Training PBs Affect Recovery, Performance, Health, Physical Fitness, and Career in Competitive Athletes?

**Figure 4:**
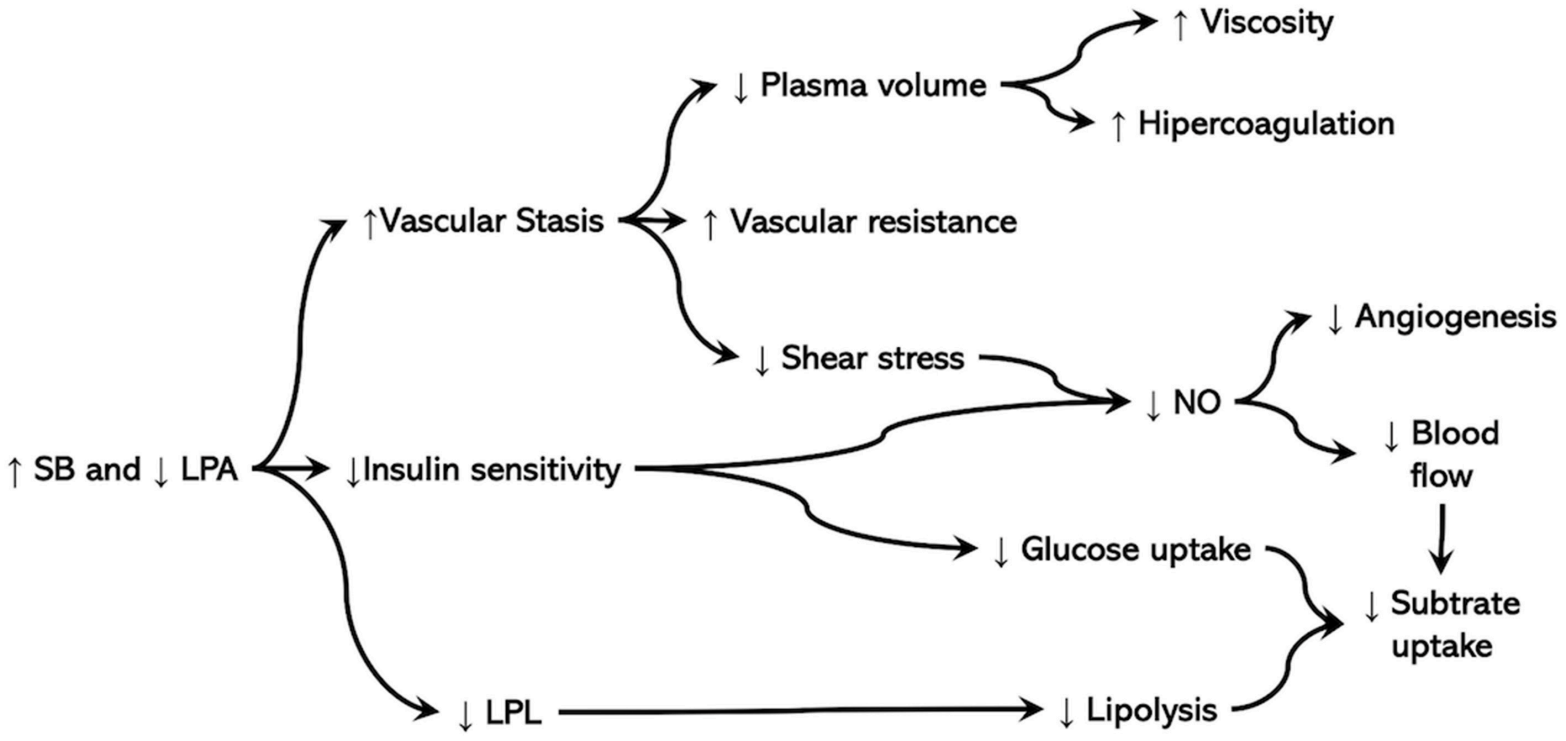
SB, Sedentary behavior; LPA, light-intensity physical activity; LPL, lipoprotein lipase; NO, nitric oxide. ↑, indicates an increase in the amount or activity; ↓, indicates a decrease in the amount or activity. Prolonged and uninterrupted SB and reduced physical activity during the day, mainly represented by LPA, may potentially affect recovery through the vascular and metabolic mechanisms indicated in the figure.

Only two studies associated off-training PBs in competitive athletes with variables associated with health or recovery. Júdice et al. (2014) reported sitting time was positively associated with total and trunk adiposity in different groups of elite athletes, while weight class sports athletes, despite sustaining lower training volume, showed the highest values of sitting time and the strongest association between sitting time and adiposity. This association may cause surprise because training is considered an activity with high energy expenditure and elite athletes should train enough to maintain an excellent body composition. Indeed, the sample in the study into consideration reported a weekly training time that was far above the MVPA recommendations for the general population or the highest active group in previous studies that also found an association between fatness and SB (Vandelanotte et al., 2009). However, the lowest weekly training time was reported by weight class sports participants who also showed the strongest association with adiposity, while the two sports groups that performed higher weekly training times (>20 h/week) showed no association. Then, higher levels of MVPA may compensate for the effects of time spent in SB. On the other hand, a higher amount of training in some categories of athletes may “replace” SB, while the ones with lower weekly training time may have a larger amount of leisure time that can be spent in low energy expenditure activities, especially during a preparation period for a competition. However, the energy balance alone cannot be sufficient to explain the positive association between SB and fat mass in athletes. Indeed, both glucose and lipid metabolisms are affected by SB and lack of PA, even in active people and when matching caloric intake for the reduction in energy expenditure or basal metabolism (Bergouignan et al., 2009; Stephens et al., 2011). Notably, the reduction in insulin sensitivity, lead to lower nitric oxide production and release from endothelial cells, which ends with reduced blood flow to the skeletal muscle (Wagenmakers et al., 2016). It is also possible that athletes involved in weight class sports have less energy due to energy restriction practices and for this reason are more prone to engage in SB. There is evidence that the homeostatic system tries to preserve energy to compensate both high training energy expenditure and low energy intake, reducing consciously or unconsciously SPA (Garland et al., 2011). Moreover, a negative energy balance may also affect physiological functions to preserve energy by reducing the calories spent from the immune and reproductive systems (Pontzer et al., 2015). As a consequence, both low testosterone levels (Trexler et al., 2014), especially in combat sports, and immune response (Ghaemi et al., 2014) may impair performance and recovery in athletes when they underwent weight management. Immune function affects behavior, including movement: elevated circulating cytokines induce a set of behaviors referred to as “sickness” behavior, which involves mood, and behavior changes (e.g., excessive sleep, asthenia, lack of appetite) that in normal conditions support resolution of systemic inflammation but in athletes with poor rest, injuries, and competitive pressure may lead to the overtraining syndrome (Smith, 2000) Although not yet demonstrated, measuring PBs may represent a valid tool for monitoring the risk of overtraining as well as recovery after training or injuries. Preliminary evidence in this direction emerge from one of the studies included in this scoping review which found an association between the levels of PA and the course of recovery following sport-related concussion (Sufrinko et al., 2018). Sport-related concussion itself has a significant neuroinflammatory component which could explain both the association between PA levels and post-concussion recovery as well as the highest sedentary time in weight class sports, represented by combat sports in the study of Júdice et al. (2014), although in the absence of evident concussion. It is important to underline that sitting time in that study was assessed by questionnaires. Then, although they indicated an association that was somewhat surprising considering the investigated population, future studies need to investigate the association between off-training PBs and body composition, vascular adaptations, tissue repair after training and competition, as well as with physiological and psychological indices of recovery. Furthermore, the putative mechanisms (Figure 4) should be extensively investigated to better address intervention protocols during off-training periods in athletes to promote repair and recovery.

### Can Off-Training Physical Behaviors be Considered as a Recovery Strategy?

No studies in our search used off-training PBs while awake as a strategy to improve recovery, training adaptation or performance. Then, unfortunately, current literature does not allow us to know if an active off-training lifestyle can offset fatigue, accelerate the return to “baseline” body function and to the reference performance levels, or even accelerate tissue repair. The following discussion is therefore based on evidence from studies examining recovery strategies other than off-training PBs. It can be hypothesized that if active recovery can do it, then, a certain amount of SPA should be beneficial for recovery. However, active recovery does not seem to be a universal solution to foster regeneration: in energetic terms, active recovery seems to be superior to passive recovery when performance must be repeated in a short period (< 30 min) and when exercise involves a significant anaerobic contribution (Franchini et al., 2003; Heyman et al., 2009). Active recovery favors an earlier decrease in lactatemia (Bangsbo et al., 1994) and it allows a faster return to resting pH values than passive recovery (Fairchild et al., 2003). This reduces exercise-induced acidosis, promotes co-transport of lactate and H^+^ ions from intramuscular medium and the blood, and then it should preserve neuromuscular function during anaerobic tasks that are performed close to each other. Interestingly, active recovery involving large muscle mass is beneficial also when the event does not involve the same muscle groups that exerted the recovery task (climbing vs. pedaling) (Heyman et al., 2009). Furthermore, aerobic training involving a large muscle mass determines higher circulating levels of VEGF (Izzicupo et al., 2017) that can improve local blood flow in resting muscle. Notably, oxygen availability (McMahon and Jenkins, 2002) and aerobic fitness (Buchheit and Ufland, 2011) are crucial for a fast phosphocreatine resynthesis between short-term events such as repeated sprints. In contrast, depleted glycogen restoration seems to benefit more when using passive recovery (Choi et al., 1994), probably because active recovery further mobilizes glycogen stores. In term of recovery from muscle damage rather than energy restoration, results are contrasting: several studies focused on active recovery after training with both better (Gill, 2006; Tufano et al., 2012) and worse (Sherman et al., 1984; Suzuki, 2004) effects when compared to passive recovery. Overall, active recovery seems to be more effective after eccentrically-based activity (Tufano et al., 2012) and when duration is very short (Gill, 2006). On the other hand, active recovery in the days following an exhaustive competition (i.e., marathon race) seems to be detrimental (Sherman et al., 1984). These results suggest that the amount, intensity, and timing of active recovery are crucial, and, in some cases, it can be better to simply rest rather than perform prolonged active recovery. Probably, while sitting too much may impair local blood flow to the muscle and metabolic efficiency, passive rest can simply be necessary to allocate the energy to the anabolic processes taking place after training and during recovery. Under this point of view, also the intensity of the activities performed during recovery can be important. Several studies suggested that LPA is sufficient to promote positive effects on health (Chastin et al., 2018), however, our knowledge about physiological adaptations to low-intensity work as a recovery strategy in athletes is very limited to date. Hypothetically, sitting interruption and LPA, both implemented off-training and during daily activities, should be considered a promising approach to the problem. Since few minutes seems to be better than longer active recovery interventions, just frequent interruptions of SB through LPA can both promote the positive effects of active recovery, on the one hand, and counteract the side effect of prolonged sitting, on the other one. Furthermore, LPA may be implemented after the main meals to avoid the detrimental metabolic effects of post-prandial prolonged sitting (Thorp et al., 2014) as well as during off-season or after suffering an injury, because some athletes may be very inactive in these phases, compromising the ability to maintain specific fitness components and return to optimal conditions. The activity levels of athletes during these conditions, though, need to be investigated in the future using reliable and standardized methods.

However, at this point, an important consideration must be made regarding the nature of rest and SB: although rest can be performed in a sedentary way, it should be no more or less than the body needs to recover, while, implicitly, SB is harmful because it is over-prolonged. This distinction is a very difficult challenge that, in our opinion, scientific research will have to face in the next years by designing intervention studies in which PBs is modified via education (or other means) to improve athletes' lifestyles and habits.

This is a scoping review to present and mapping the current state of evidence specific to off-training PBs while awake in competitive athletes. The main limitation of this article is that we were not able to answer to part of the formulated scientific questions. Furthermore, both objective and subjective PBs measurement methods were included into the study. Although in our opinion such a choice was necessary to map current understanding on PBs in competitive athletes, the risk of collapsing data that are not directly comparable is high. Nonetheless, this article suggests that at the moment the athlete's lifestyle management is based on nothing more than simple recommendations that are not based in scientific evidence, due to the paucity of indication relative to off-training PBs in athletes. The 3ST project aims to fill the gap in current literature, first by means of descriptive researches, secondly investigating the putative mechanisms of the eventual effects of SB and SPA on recovery and performance and finally, designing intervention studies in which PBs are modified via education (or other means) to improve athletes' lifestyles and habits. This scoping review article aims at motivating the sport scientists, practitioners and athletes to involve themselves in this new research topic that, based on the limited evidence, can be the next stage to promote better lifestyles along with longer and more successful careers. The results of the 3ST project and future researches related to this topic will be useful to go beyond anecdotal recommendation on lifestyle for athletes and, possibly, allow the implementation of a conceptual model on how athletes should pursue PBs to improve recovery and performance, as well as specific 24-h hygiene recommendations on PBs for athletes.

## Author Contributions

PI and FYN: conceptualization and writing-original draft. PI and FFR: data curation and formal analysis. PI, FYN, FFR, and EK: methodology. PI, BG, and FYN: investigation. BG and ADB: visualization. PI, ADB, BG, FFR, EK, and FYN: writing-review and editing. ADB: fundings.

### Conflict of Interest Statement

The authors declare that the research was conducted in the absence of any commercial or financial relationships that could be construed as a potential conflict of interest.

## References

[B1] Ala-Kitula A. Peltonen J. Finni T. Linnamo V. (2019). Physical activity on days with and without soccer practice in 12-13-year-old boys. Sci. Med. Football. 1–6. 10.1080/24733938.2018.1562276 [Epub ahead of print].

[B2] Bangsbo J. Graham T. Johansen L. Saltin B. (1994). Muscle lactate metabolism in recovery from intense exhaustive exercise: impact of light exercise. J. Appl. Physiol. 77, 1890–1895. 10.1152/jappl.1994.77.4.1890 7836214

[B3] Bergouignan A. Trudel G. Simon C. Chopard A. Schoeller D. A. Momken I.. (2009). Physical inactivity differentially alters dietary oleate and palmitate trafficking. Diabetes 58, 367–376. 10.2337/db08-0263 19017764 PMC2628610

[B4] Bishop M. Astolfi M. Padegimas E. DeLuca P. Hammoud S. (2017). Venous thromboembolism within professional American sport leagues. Orthopaed. J. Sports Med. 5:232596711774553. 10.1177/2325967117745530 29318176 PMC5753956

[B5] Biswas A. Oh P. I. Faulkner G. E. Bajaj R. R. Silver M. A. Mitchell M. S.. (2015). Sedentary time and its association with risk for disease incidence, mortality, and hospitalization in adults: a systematic review and meta-analysis. Ann. Int. Med. 162, 123–132. 10.7326/M14-1651 25599350

[B6] Blasio A. D. Donato F. D. Santo S. D. Bucci I. Izzicupo P. Baldassarre A. D.. (2018). Aerobic physical exercise and negative compensation of non-exercise physical activity in post-menopause: a pilot study. J. Sports Med. Phys. Fitness 58, 1497–1508. 10.23736/S0022-4707.17.07320-0 28597615

[B7] Buchheit M. Ufland P. (2011). Effect of endurance training on performance and muscle reoxygenation rate during repeated-sprint running. Eur. J. Appl. Physiol. 111, 293–301. 10.1007/s00421-010-1654-9 20872150

[B8] Chaput J.-P. Carson V. Gray C. Tremblay M. (2014). Importance of all movement behaviors in a 24 hour period for overall health. Int. J. Environ. Res. Public Health 11, 12575–12581. 10.3390/ijerph111212575 25485978 PMC4276632

[B9] Chastin S. F. M. De Craemer M. De Cocker K. Powell L. Van Cauwenberg J. Dall P.. (2018). How does light-intensity physical activity associate with adult cardiometabolic health and mortality? Systematic review with meta-analysis of experimental and observational studies. Br. J. Sports Med. 53, 370–376. 10.1136/bjsports-2017-097563 29695511 PMC6579499

[B10] Choi D. Cole K. J. Goodpaster B. H. Fink W. J. Costill D. L. (1994). Effect of passive and active recovery on the resynthesis of muscle glycogen. Med. Sci. Sports Exerc. 26, 992–996. 10.1249/00005768-199408000-00010 7968434

[B11] Clemente F. M. Nikolaidis P. T. Martins F. M. L. Mendes R. S. (2016). Weekly physical activity patterns of university students: are athletes more active than non-athletes? SpringerPlus 5:1808. 10.1186/s40064-016-3508-3 27812448 PMC5069237

[B12] Colley R. C. Michaud I. Garriguet D. (2018). Reallocating time between sleep, sedentary and active behaviours: associations with obesity and health in Canadian adults. Health Rep. 29, 3–13. Available online at: https://www.statcan.gc.ca 29668028

[B13] Condello G. Ling F. C. M. Bianco A. Chastin S. Cardon G.. (2016). Using concept mapping in the development of the EU-PAD framework (EUropean-Physical Activity Determinants across the life course): a DEDIPAC-study. BMC Public Health 16:1145. 10.1186/s12889-016-3800-8 27825370 PMC5101801

[B14] Costa J. A. Brito J. Nakamura F. Y. Oliveira E. M. Costa O. P. Rebelo A. N. (2018). Does night-training load affect sleep patterns and nocturnal cardiac autonomic activity in high-level female soccer players? Int. J. Sports Physiol. Perf. 20, 1–26. 10.1123/ijspp.2018-0652 30569771

[B15] Di Blasio A. Ripari P. Bucci I. Di Donato F. Izzicupo P. D'Angelo E.. (2012). Walking training in postmenopause: effects on both spontaneous physical activity and training-induced body adaptations. Menopause J. North Am. Menopause Soc. 19, 23–32. 10.1097/gme.0b013e318223e6b3 21993080

[B16] Dyrstad S. M. Hansen B. H. Holme I. M. Anderssen S. A. (2014). Comparison of self-reported versus accelerometer-measured physical activity. Med. Sci. Sports Exerc. 46, 99–106. 10.1249/MSS.0b013e3182a0595f 23793232

[B17] Ekelund U. Steene-Johannessen J. Brown W. J. Fagerland M. W. Owen N. Powell K. E.. (2016). Does physical activity attenuate, or even eliminate, the detrimental association of sitting time with mortality? A harmonised meta-analysis of data from more than 1 million men and women. Lancet 388, 1302–1310. 10.1016/S0140-6736(16)30370-1 27475271

[B18] Exel J. Mateus N. Abrantes C. Leite N. Sampaio J. (2019). Physical activity and sedentary behavior in amateur sports: master athletes are not free from prolonged sedentary time. Sport Sci. Health. 10.1007/s11332-019-00527-3 [Epub ahead of print].

[B19] Exel J. Mateus N. Travassos B. Gonçalves B. Gomes I. Leite N.. (2018). Off-training levels of physical activity and sedentary behavior in young athletes: preliminary results during a typical week. Sports 6:141. 10.3390/sports6040141 30404165 PMC6316694

[B20] Fairchild T. J. Armstrong A. A. Rao A. Liu H. Lawrence S. Fournier P. A. (2003). Glycogen synthesis in muscle fibers during active recovery from intense exercise. Med. Sci. Sports Exerc. 35, 595–602. 10.1249/01.MSS.0000058436.46584.8E 12673142

[B21] Fairclough S. J. Beighle A. Erwin H. Ridgers N. D. (2012). School day segmented physical activity patterns of high and low active children. BMC Public Health 12:406. 10.1186/1471-2458-12-406 22672654 PMC3434066

[B22] Fowler P. M. Knez W. Crowcroft S. Mendham A. E. Miller J. Sargent C.. (2017). Greater effect of east versus west travel on jet lag, sleep, and team sport performance. Med. Sci. Sports Exerc. 49, 2548–2561. 10.1249/MSS.0000000000001374 28719491

[B23] Franchini E. Takito M. Y. Nakamura F. Y. Matsushigue K. A. Peduti M. A. (2003). Effects of recovery type after a judo combat on blood lactate removal and on performance in an intermittent anaerobic task. J. Sports Med. Phys. Fitness 4, 424–431. 10.1007/s00421-009-1134 14767401

[B24] Freedson P. (2018). Using wearable devices to assess physical behavior in epidemiologic research. J. Meas. Phys. Behav. 1, 49–50. 10.1123/jmpb.2018-0029

[B25] Garatachea N. Santos-Lozano A. Sanchis-Gomar F. Fiuza-Luces C. Pareja-Galeano H. Emanuele E.. (2014). Elite athletes live longer than the general population: a meta-analysis. Mayo Clinic Proceed. 89, 1195–1200. 10.1016/j.mayocp.2014.06.004 25128074

[B26] Garland T. Schutz H. Chappell M. A. Keeney B. K. Meek T. H. Copes L. E.. (2011). The biological control of voluntary exercise, spontaneous physical activity and daily energy expenditure in relation to obesity: human and rodent perspectives. J. Exp. Biol. 214, 206–229. 10.1242/jeb.048397 21177942 PMC3008631

[B27] Ghaemi J. Rashidlamir A. Hosseini S. R. A. Mohammad Rahimi G. R. (2014). The effect of rapid and gradual weight loss on some hematological parameters in trained wrestlers. Int. J. Wrestling Sci. 4, 37–41. 10.1080/21615667.2014.954497

[B28] Gill N. D. (2006). Effectiveness of post-match recovery strategies in rugby players. Br. J. Sports Med. 40, 260–263. 10.1136/bjsm.2005.022483 16505085 PMC2491972

[B29] Graf C. Ferrari N. Beneke R. Bloch W. Eiser S. Koch B. (2017). Empfehlungen für körperliche Aktivität und Inaktivität von Kindern und Jugendlichen – Methodisches Vorgehen, Datenbasis und Begründung. Das Gesundheitswesen 79(S 01), S11–9. 10.1055/s-0042-123701 28399581

[B30] Green A. Cohen-Zion M. Haim A. Dagan Y. (2017). Evening light exposure to computer screens disrupts human sleep, biological rhythms, and attention abilities. Chronobiol. Int. 34, 855–865. 10.1080/07420528.2017.1324878 28548897

[B31] Heyman E. De Geus B. Mertens I. Meeusen R. (2009). Effects of four recovery methods on repeated maximal rock climbing performance. Med. Sci. Sports Exerc. 41, 1303–1310. 10.1249/MSS.0b013e318195107d 19461534

[B32] Hildebrand M. VAN Hees V. T. Hansen B. H. Ekelund U. (2014). Age group comparability of raw accelerometer output from wrist- and hip-worn monitors. Med. Sci. Sports Exerc. 46, 1816–1824. 10.1249/MSS.0000000000000289 24887173

[B33] Howard B. J. Fraser S. F. Sethi P. Cerin E. Hamilton M. T. Owen N.. (2013). Impact on hemostatic parameters of interrupting sitting with intermittent activity. Med. Sci. Sports Exerc. 45, 1285–1291. 10.1249/MSS.0b013e318285f57e 23439415

[B34] Izzicupo P. D'Amico M. A. Bascelli A. Di Fonso A. D'Angelo E. Di Blasio A.. (2012). Walking training affects dehydroepiandrosterone sulfate and inflammation independent of changes in spontaneous physical activity. Menopause J. North Am. Menopause Soc. 20, 455–63. 10.1097/gme.0b013e31827425c9 23250080

[B35] Izzicupo P. D'Amico M. A. Di Blasio A. Napolitano G. Di Baldassarre A. Ghinassi B. (2017). Nordic walking increases circulating VEGF more than traditional walking training in postmenopause. Climacteric 20, 533–539. 10.1080/13697137.2017.1366979 28920458

[B36] Jones M. J. Dawson B. Gucciardi D. F. Eastwood P. R. Miller J. Halson S. L.. (2019). Evening electronic device use and sleep patterns in athletes. J. Sports Sci. 37, 864–870. 10.1080/02640414.2018.1531499 30326782

[B37] Júdice P. B. Silva A. M. Magalhães J. P. Matias C. N. Sardinha L. B. (2014). Sedentary behaviour and adiposity in elite athletes. J Sports Sci. 32, 1760–1767. 10.1080/02640414.2014.926382 24915288

[B38] Kettunen J. A. Kujala U. M. Kaprio J. Bäckmand H. Peltonen M. Eriksson J. G.. (2015). All-cause and disease-specific mortality among male, former elite athletes: an average 50-year follow-up. Br. J. Sports Med. 49, 893–897. 10.1136/bjsports-2013-093347 25183628

[B39] Lemez S. Baker J. (2015). Do elite athletes live longer? A systematic review of mortality and longevity in elite athletes. Sports Med. Open 1:16. 10.1186/s40798-015-0024-x 26301178 PMC4534511

[B40] McCracken H. Dogra S. (2018). Sedentary time in male and female masters and recreational athletes aged 55 and older. J. Aging Phys. Activity 26, 121–127. 10.1123/japa.2016-0324 28595024

[B41] McGregor D. Carson V. Palarea-Albaladejo J. Dall P. Tremblay M. Chastin S. (2018). Compositional analysis of the associations between 24-h movement behaviours and health indicators among adults and older adults from the Canadian health measure survey. Int. J. Environ. Res. Public Health 15:1779. 10.3390/ijerph15081779 30126215 PMC6121426

[B42] McGuigan M. (2017). Monitoring Training and Performance in Athletes. Champaign, IL: Human Kinetics.

[B43] McMahon S. Jenkins D. (2002). Factors affecting the rate of phosphocreatine resynthesis following intense exercise. Sports Med. 32, 761–784. 10.2165/00007256-200232120-00002 12238940

[B44] Meyering C. Howard T. (2004). Hypercoagulability in athletes. Curr. Sports Med. Rep. 3, 77–83. 10.1249/00149619-200404000-00005 14980135

[B45] Ministry of the Education Culture Finland (2016). Joy, Play and Doing Together–Recommendations for Physical Activity in Early Childhood. Retrieved from http://urn.fi/URN:ISBN:978-952-263-413-9

[B46] Mottola M. F. Davenport M. H. Ruchat S.-M. Davies G. A. Poitras V. J. Gray. (2018). 2019 Canadian guideline for physical activity throughout pregnancy. Br. J. Sports Med. 52, 1339–1346. 10.1136/bjsports-2018-100056 30337460

[B47] Nedelec M. Aloulou A. Duforez F. Meyer T. Dupont G. (2018). The variability of sleep among elite athletes. Sports Med Open 4:34. 10.1186/s40798-018-0151-2 30054756 PMC6063976

[B48] New Zealand Ministry of Health. Sit Less, Move More, Sleep Well: Active Play Guidelines for under-fives Available online at: https://www.health.govt.nz/publication/sit-less-move-more-sleep-well-active-play-guidelines-under-fives (accessed: February 6, 2019).,

[B49] Okely A. D. Ghersi D. Hesketh K. D. Santos R. Loughran S. P. Cliff D. P.. (2017). A collaborative approach to adopting/adapting guidelines-the Australian 24-hour movement guidelines for the early years (Birth to 5 years): an integration of physical activity, sedentary behavior, and sleep. BMC Public Health 17:869. 10.1186/s12889-017-4867-6 29219094 PMC5773882

[B50] Padilla J. Fadel P. J. (2017). Prolonged sitting leg vasculopathy: contributing factors and clinical implications. Am. J. Physiol. 313, H722–H728. 10.1152/ajpheart.00326.2017 28733451 PMC5668607

[B51] Pedersen B. K. Saltin B. (2015). Exercise as medicine - evidence for prescribing exercise as therapy in 26 different chronic diseases. Scandinavian J. Med. Sci. Sports 25, 1–72. 10.1111/sms.12581 26606383

[B52] Peters M. D. J. Godfrey C. M. Khalil H. McInerney P. Parker D. Soares C. B. (2015). Guidance for conducting systematic scoping reviews. Int. J. Evid. Based Healthcare 13, 141–146. 10.1097/XEB.0000000000000050 26134548

[B53] Pfeifer K. Geidl W. (2017). Bewegungsempfehlungen für Erwachsene mit einer chronischen Erkrankung–Methodisches Vorgehen, Datenbasis und Begründung. Das Gesundheitswesen 79(S01), S29–S35. 10.1055/s-0042-123699 28399583

[B54] Piercy K. L. Troiano R. P. Ballard R. M. Carlson S. A. Fulton J. E. Galuska D. A.. (2018). The physical activity guidelines for Americans. JAMA 320, 2020–2028. 10.1001/jama.2018.14854 30418471 PMC9582631

[B55] Pontzer H. Raichlen D. A. Wood B. M. Emery Thompson M. Racette S. B. Mabulla A. Z. P. (2015). Energy expenditure and activity among Hadza hunter-gatherers: Hadza energetics and activity. Am. J. Hum. Biol. 27, 628–637. 10.1002/ajhb.22711 25824106

[B56] Sedentary Behaviour Research Network (2012). Letter to the Editor: Standardized use of the terms “sedentary” and “sedentary behaviours.” Appl. Physiol. Nutr. Metab. 37, 540–542. 10.1139/h2012-024 22540258

[B57] Sharma S. (2003). Physiological society symposium-the Athlete's heart: athlete's heart - effect of age, sex, ethnicity and sporting discipline. Exp. Physiol. 88, 665–669. 10.1113/eph8802624 12955167

[B58] Sherman W. M. Armstrong L. E. Murray T. M. Hagerman F. C. Costill D. L. Staron R. C.. (1984). Effect of a 42.2-km footrace and subsequent rest or exercise on muscular strength and work capacity. J. Appl. Physiol. 57, 1668–1673. 10.1152/jappl.1984.57.6.1668 6511541

[B59] Smith L. L. (2000). Cytokine hypothesis of overtraining: a physiological adaptation to excessive stress? Med. Sci. Sports Exerc. 32, 317–331. 10.1097/00005768-200002000-00011 10694113

[B60] Sperlich B. Becker M. Hotho A. Wallmann-Sperlich B. Sareban M. Winkert K.. (2017). Sedentary behavior among national elite rowers during off-training—a pilot study. Front. Physiol. 8:655. 10.3389/fphys.2017.00655 28979208 PMC5611419

[B61] Stephens B. R. Granados K. Zderic T. W. Hamilton M. T. Braun B. (2011) Effects of 1 day of inactivity on insulin action in healthy men women: interaction with energy intake. Metabolism 60, 941–949. 10.1016/j.metabol.2010.08.014 21067784

[B62] Sufrinko A. M. Howie E. K. Elbin R. J. Collins M. W. Kontos A. P. (2018). A preliminary investigation of accelerometer-derived sleep and physical activity following sport-related concussion. J. Head Trauma Rehabilitat. 33, E64–E74. 10.1097/HTR.0000000000000387 29601343

[B63] Suzuki M. (2004). Effect of incorporating low intensity exercise into the recovery period after a rugby match. Br. J. Sports Med. 38, 436–440. 10.1136/bjsm.2002.004309 15273179 PMC1724892

[B64] Thorp A. A. Kingwell B. A. Sethi P. Hammond L. Owen N. Dunstan D. W. (2014). Alternating bouts of sitting and standing attenuate postprandial glucose responses. Med. Sci. Sports Exerc. 46, 2053–2061. 10.1249/MSS.0000000000000337 24637345

[B65] Tremblay M. S. Aubert S. Barnes J. D. Saunders T. J. Carson V. Latimer-Cheung A. M.. (2017a). Sedentary Behavior Research Network (SBRN)–terminology consensus project process and outcome. Int. J. Behav. Nutr. Phys. Activity 14:75. 10.1186/s12966-017-0525-8 28599680 PMC5466781

[B66] Tremblay M. S. Carson V. Chaput J.-P. Connor Gorber S. Dinh T. Duggan M. (2016). Canadian 24-hour movement guidelines for children and youth: an integration of physical activity, sedentary behaviour, and sleep. Appl. Physiol. Nutr. Metab. 41(Suppl. 3), S311–S327. 10.1139/apnm-2016-0151 27306437

[B67] Tremblay M. S. Chaput J.-P. Adamo K. B. Aubert S. Barnes J. D. Choquette L.. (2017b). Canadian 24-hour movement guidelines for the early years (0–4 years): an integration of physical activity, sedentary behaviour, and sleep. BMC Public Health 17:874. 10.1186/s12889-017-4859-6 29219102 PMC5773896

[B68] Trexler E. T. Smith-Ryan A. E. Norton L. E. (2014). Metabolic adaptation to weight loss: implications for the athlete. J. Int. Soc. Sports Nutr. 11:7. 10.1186/1550-2783-11-7 24571926 PMC3943438

[B69] Tufano J. J. Brown L. E. Coburn J. W. Tsang K. K. W. Cazas V. L. LaPorta J. W. (2012). Effect of aerobic recovery intensity on delayed-onset muscle soreness and strength. J. Strength Cond. Res. 26, 2777–2782. 10.1519/JSC.0b013e3182651c06 22739325

[B70] Vandelanotte C. Sugiyama T. Gardiner P. Owen N. (2009). Associations of leisure-time internet and computer use with overweight and obesity, physical activity and sedentary behaviors: cross-sectional study. J. Med. Int. Res. 11:e28. 10.2196/jmir.1084 19666455 PMC2762849

[B71] Vitale J. A. Banfi G. Galbiati A. Ferini-Strambi L. La Torre A. (2019a). Effect of a night game on actigraphy-based sleep quality and perceived recovery in top-level volleyball athletes. Int. J. Sports Physiol. Perf. 14, 265–269. 10.1123/ijspp.2018-0194 30040006

[B72] Vitale J. A. Banfi G. Sias M. La Torre A. (2019b). Athletes' rest-activity circadian rhythm differs in accordance with the sport discipline. Chronobiol. Int. 36, 578–586. 10.1080/07420528.2019.1569673 30760036

[B73] Wagenmakers A. J. M. Strauss J. A. Shepherd S. O. Keske M. A. Cocks M. (2016). Increased muscle blood supply and transendothelial nutrient and insulin transport induced by food intake and exercise: effect of obesity and ageing: transendothelial transport of nutrients and insulin to muscle fibres. J. Physiol. 594, 2207–2222. 10.1113/jphysiol.2014.284513 25627798 PMC4933113

[B74] Weiler R. Aggio D. Hamer M. Taylor T. Kumar B. (2015). Sedentary behaviour among elite professional footballers: health and performance implications. BMJ Open Sport Exerc. Med. 1:e000023. 10.1136/bmjsem-2015-000023 27110383 PMC4838833

[B75] Young F. L. S. Leicht A. S. (2011). Short-term stability of resting heart rate variability: influence of position and gender. Appl. Physiol. Nutr. Metab. 36, 210–218. 10.1139/h10-103 21609282

