# Supplementary material for: Can Off-Training Physical Behaviors Influence Recovery in Athletes? A Scoping Review
Source: Front Physiol. 2019 Apr 30;10:448. doi: 10.3389/fphys.2019.00448 (PMC6503646; doi:10.3389/fphys.2019.00448)
Supplement: Supplementary file 1 [file Table_1.DOCX]

Search syntax

MEDLINE

(player*[Title/Abstract] OR athlete*[Title/Abstract] OR amateur*[Title/Abstract] OR professional*[Title/Abstract] OR elite[Title/Abstract]) AND (self-reported time[Title/Abstract] OR questionnaire* [Title/Abstract] OR acceleromet*[Title/Abstract]) AND (highly trained[Title/Abstract] OR injur*[Title/Abstract] OR recreational[Title/Abstract] OR young adult*[Title/Abstract] OR master*[Title/Abstract] OR young athlete*[Title/Abstract] OR adolescent*[Title/Abstract] OR youth[Title/Abstract] OR non-athlet*[Title/Abstract]) AND (sitting[Title/Abstract] OR physical activit*[Title/Abstract] OR sedentar*[Title/Abstract] OR recovery[Title/Abstract] OR performance[Title/Abstract])

SCOPUS

( TITLE-ABS-KEY ( player* ) OR TITLE-ABS-KEY ( athlete* ) TITLE-ABS-KEY ( amateur* ) OR TITLE-ABS-KEY ( professional * ) OR TITLE-ABS-KEY ( elite* ) ) AND ( TITLE-ABS-KEY ( self-reported AND time ) OR TITLE-ABS-KEY ( questionnaire* ) OR TITLE-ABS-KEY (acceleromet*) ) AND ( TITLE-ABS-KEY ( highly AND trained ) OR TITLE-ABS-KEY ( injur* ) OR TITLE-ABS-KEY ( recreational ) OR TITLE-ABS-KEY ( young AND adult*) OR TITLE-ABS-KEY ( master* ) OR TITLE-ABS-KEY ( young AND athlete* ) OR TITLE-ABS-KEY ( adolescent* ) OR TITLE-ABS-KEY ( youth ) OR TITLE-ABS-KEY ( non-athlet* ) ) AND ( TITLE-ABS-KEY ( sedent* ) OR TITLE-ABS-KEY ( physical AND activit* ) OR TITLE-ABS-KEY ( sitting ) OR TITLE-ABS-KEY ( performance ) OR TITLE-ABS-KEY ( recovery ) )

ISI WOS

(TITLE: (Player*) OR TITLE: (athlete*) OR TITLE: (amateur*) OR TITLE: (professional*) OR TITLE: (elite)) AND (TITLE: (self-reported time) OR TITLE: (questionnaire*) OR TITLE: (accelerometer*)) AND (TITLE: (highly trained) OR TITLE: (injur*) OR TITLE: (recreational) OR TITLE: (young adult*) OR TITLE: (master) OR TITLE: (young athlete*) OR TITLE: (non-athlet*) OR TITLE: (adolescent*) OR TITLE: (youth)) AND (TITLE: (sitting) OR TITLE: (physical activit*) OR TITLE: (sedentar*) OR TITLE: (recovery) OR TITLE: (performance))
